# Aerosols generation using Er,Cr:YSGG laser compared to rotary instruments in conservative dentistry: A preliminary study

**DOI:** 10.4317/jced.57731

**Published:** 2021-01-01

**Authors:** Haitham Abdelkarim-Elafifi, Cristina Arnabat-Artés, Isabel Parada-Avendaño, Marina Polonsky, Josep Arnabat-Domínguez

**Affiliations:** 1 DDS, MSc. Faculty of Medicine and Health Sciences, University of Barcelona. Barcelona, Spain. Master of Laser in Dentistry (EMDOLA) University of Barcelona. Spain; 2 MD, MSc. Master degree in analysis and design in clinical investigation, University of Barcelona, Spain; 3 DDS, MSc. Canadian Dental Laser institute. Ottawa, Canada; 4 MD, DDS, MSc, PhD. Researcher at the Idibell institute. Barcelona, Spain

## Abstract

**Background:**

In restorative dentistry, the use of high-speed air turbine, which generates aerosols, can be associated with the transmission of airborne diseases. New laser technologies could be useful in reducing the amount of aerosols, but there is a lack of scientific research on this topic.

**Material and Methods:**

This is a descriptive study to analyze the amount of aerosols produced after class I cavity preparation using high-speed air turbine (group 1) and Er,Cr:YSGG laser with two different parameters (groups 2 and 3). Fluorescein dye was incorporated into the coolant reservoir in order to visualize the production of aerosols during each procedure. Tooth preparation was performed in a typodont with human lower molar tooth under rubber dam isolation. The procedure was carried out in a transparent plastic box to avoid aerosols dispersion. Sixteen grade I cellulose filter discs were distributed along the surfaces of the box. The area contaminated with aerosols in the filters was measured using ultraviolet illumination.

**Results:**

In group 1, the contaminated surface area covered with fluorescein dye reached 77.3% (1349 cm2) of the total; in group 2 (laser with 80% water) we observed 7.3% (128 cm2) and in group 3 (laser with 40% water) it was 3.8% (68 cm2). The reduction in water parameter from 80% to 40% coincided with 48% reduction of the contaminated area on the filter discs. Focusing on the surfaces of the box, we noted that the mean contamination on the left side was more than on the right side in all three experimental groups. In group 1 using air turbine, we measured a mean of 102.6[±7.5 SD]cm2 on the left side, compared to 70.6[±32.3 SD]cm2 on the right side. In laser groups 2 and 3, a mean of 12.8[±14.9 SD]cm2 and 6.8 [±5.7SD]cm2, respectively, was described on the left surface versus 0 cm2 of surface contamination on the right surface.

**Conclusions:**

The contaminated area during the procedure of class I cavity preparation, is reduced by 70% using Er,Cr:YSGG laser compared to high-speed turbine. A slightly higher contamination was observed between laser groups with 80% versus 40% water. The use of Er,Cr:YSGG laser in restorative dentistry can be a valid treatment alternative to reduce aerosols production compared to conventional high-speed rotary instruments.

** Key words:**Er,Cr:YSGG laser, Aerosols, SARS-CoV-2, Rotary instruments, conservative dentistry.

## Introduction

The pandemic triggered by the novel coronavirus causing Severe Acute Respiratory Syndrome (SARS-CoV-2) started in December 2019 in Wuhan, China (1), affecting 195 countries around the world. Its high transmission rate (R0 of 3.58) (2) has generated great concern in the field of dentistry.

The main symptoms of the disease include fever, dry cough, dyspnea, respiratory distress and fatigue or myalgia, as well as headache, diarrhea (3-5), hyposmia and dysgeusia (6). Recently, dermatological lesions have been reported predominantly on hands and feet, which appear mainly in children and adolescents (7).

Routes of transmission are either direct contact with oral, nasal or eye mucous membranes and via respiratory tract (coughing, sneezing and droplet inhalation) (8) or indirect via contaminated surfaces. From the analysis of conjunctival samples of confirmed cases of SARS-Cov-2 (3), eye exposure has also been demonstrated to be an effective way for the virus to enter and infect the host.

Expression of angiotensin-converting enzyme 2 (ACE2) receptor, which is used by SARS-CoV-2 as a way of cellular invasion was found in salivary glands, epithelial cells of the tongue, T cells, B cells, fibroblasts and epithelial oral mucosal lining, suggesting that the oral cavity is a possible medium for direct virus invasion and attachment (9). This explains the peak in viral load in saliva during the first week following onset of symptoms and subsequent decline (10).

Most restorative and surgical procedures in dentistry require the use of rotary instruments which have been demonstrated to generate considerable amount of splatter and aerosols (11,12). The highest amount of aerosol emission usually occurs during dental prophylaxis with ultrasonic equipment and in tooth preparation using the high-speed dental handpiece. Aerosols are suspensions of solid or liquid particles, which may contain saliva, blood elements, organic tooth particles, bacteria or viruses (13). The particle size can vary from 0.001 to >100 μm (14-16).

Van Doremalen *et al.* (17) describe the half-life of SARS-CoV-2 in aerosols being 1.2 hours (range 0.64 to 2.64 hours), 7 hours on plastic surfaces and 6 hours on stainless steel. Apart from disinfecting material and work areas, dentists must wear protective equipment including the use of special respirators like European standard Filtering Face Piece 2 (EU FFP2) and maintain a minimal distance from the patient of 35-40 cm to limit the transmission of airborne diseases, especially in procedures involving aerosols generation.

**Figure 1:**
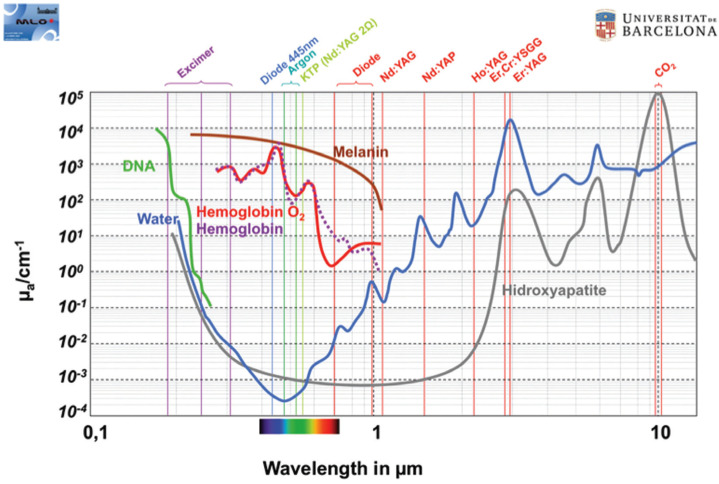
Coefficient of absorption of different wavelengths by various tissues.

The use of hard tissue lasers may be an alternative technique in many dental procedures traditionally performed by rotary instruments to reduce the amount of aerosols. Their introduction in dentistry started in the late 1980’s with advantages such as the absence of smear layer, bactericidal nature and tissue selectivity depending on the wavelength of the laser (18). For example, in restorative dentistry the Erbium Chromium: Yttrium Scandium Gallium Garnet laser (Er,Cr:YSGG) with wavelength in the mid-infrared range of the electromagnetic spectrum (2780 nm) is characterized by its energy being highly absorbed by water molecules (Fig. 1). For cavity preparations in natural teeth, the incremental pulpal temperature rise with this laser is less than 4ºC (19,20) due to the low thermal side-effect production. However, a rotatory instrument generates higher thermal side-effect because of the direct contact and friction during cutting. Therefore, higher amounts of water cooling is mandatory, leading to more aerosols generation, depending on the flow rate of the coolant (21).

The laser parameters with higher percentage of water/air allow the use of higher peak energy per pulse and lower repetition rate for faster ablation, while avoiding significant temperature increase (22). However, lower water percentage could possibly be used for similar indications.

The objective of this study is to describe the quantity of aerosols generated during class I cavity preparation performed with three different techniques: using a rotatory instrument (high-speed turbine) and Er,Cr:YSGG laser with two different parameters.

## Material and Methods

A descriptive study was carried out. Human teeth were used in each group with the following inclusion criteria: permanent lower molars extracted due to periodontal problems without structural alterations of the dentin or enamel.

This study was approved by the Ethical Committe for Clinical Research (CEIC) of the Dental Hospital of the University of Barcelona (Spain) (30/2020) and complied with the Helsinki Declaration. All patients signed the informed consent for the use of their teeth. These specimens were stored in saline solution and then refrigerated at 4ºC until mounted in a typodont.

In order to collect the aerosol particles produced by the turbine and the laser, we used fourty eight cotton cellulose discs of grade I, qualitative filters of 11 cm diameter and 0.2 mm thickness; fluorescein sodium (C20H10Na2O5), an odorless, orangered powder that is commonly used in microscopy, ophthalmology and forensic medicine.

For class I cavity preparation the following materials were used: A-DEC performer dental chair (A-DEC, Oregon United States). This chair has a refillable self contained 2-liter water bottle for the coolant spray and a conventional high-speed air turbine NSK S-Max M600L (NSK Company, Tochiji Japan) with diamond access cavity bur F0137 (Maillefer. Ballaigues Switzerland).

An Er,Cr:YSGG 2780 nm wavelength laser Express model (Biolase Technology, Irvine, CA, USA) with 600 microns Sapphire tip (MGG6. Biolase Technology, Irvine, CA, USA). An Ultraviolet light of 395 nm wavelength, was used to easily detect the flourescence material in aerosols that were collected on the filters.

**Figure 2:**
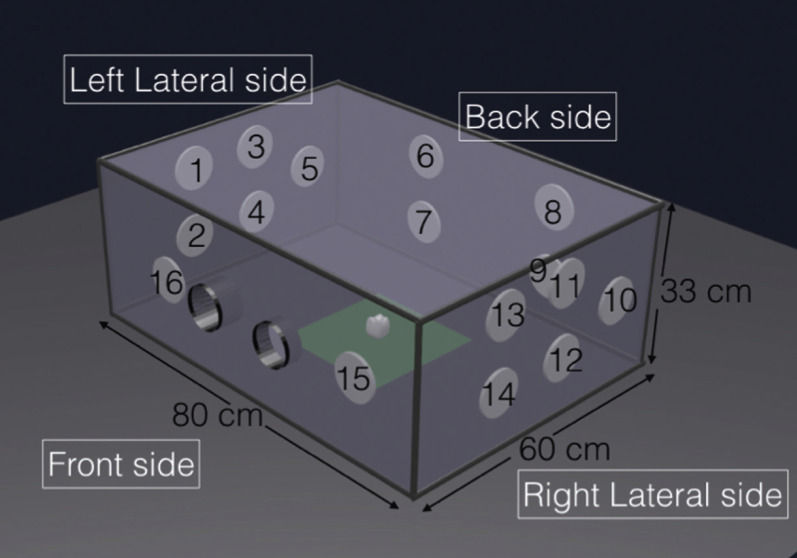
Transparent box dimension with the numbers and distribution of the filters.

The procedures were performed in a closed transparent plastic box (80 cm long x60 cm wide x 33 cm high). Two holes were made in the anterior area to insert the instruments. The typodont was placed in the centre of the box and one of the lower molar was isolated with a rubber dam and a molar clamp to simulate the clinical situation. A total of 16 filters were placed (Fig. 2): 5 filters on the right and left lateral sides, 4 filters on the posterior side and 2 filters on the anterior side. The distance from the typodont to the anterior and posterior sides of the box was 30 cm and the distance to the lateral sides was 40 cm.

Through the left hole we introduced a disposable saliva ejector for aspiration of the excess water and through the right hole a turbine or laser handpiece. We prepared the fluorescent dye placing it in the water (coolant reservoir), with the ratio 1 gram dye powder to one liter of distilled water.

**Figure 3:**
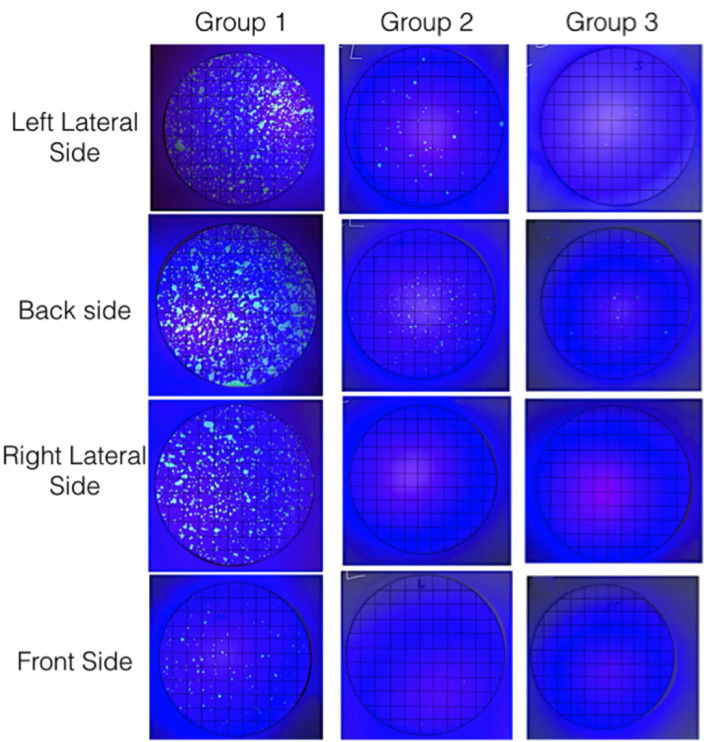
Example of a contaminated filters illuminated with UV light and gridded for area measurment, in different positions for the 3 groups.

A class I cavity was made in the tooth during 5 minutes in each group. We analyzed and took pictures with a digital camera of the fluorescent material with the help of an ultraviolet light in a dark room. With a computer program a grid template of 1x1cm2 was calibrated and super-imposed over the photo of each filter , allowing us to measure the stained area (quantitative variable meas-ured in cm2). A square was considered contaminated with a minimum stain on it (Fig. 3).

The three experimental groups were: Group 1, high speed rotary handpiece at 330,000 rpm, with water coolant at flow rate of 25 ml/min (intermediate rate). Groups 2 and 3, using Er,Cr:YSGG laser in a non contact mode at a distance of 1.5-2 mm by the parameters shown in Table 1 with water percentages of 80% and 40% respectively.

**Table 1:**
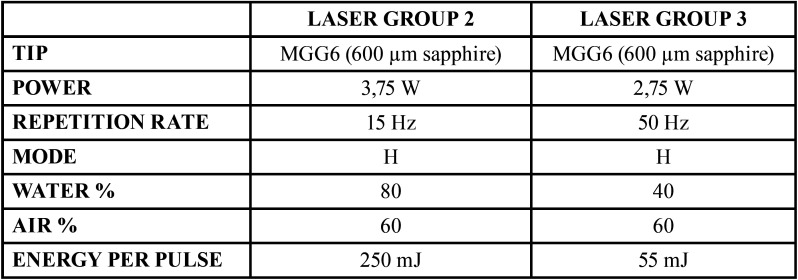
Laser groups parameters.

## Results

The variable analyzed was the contaminated area in cm2 in each filter of the 3 groups (Table 2). A total of 1744 cm2 correspond to the surface of the 16 filters.

**Table 2:**
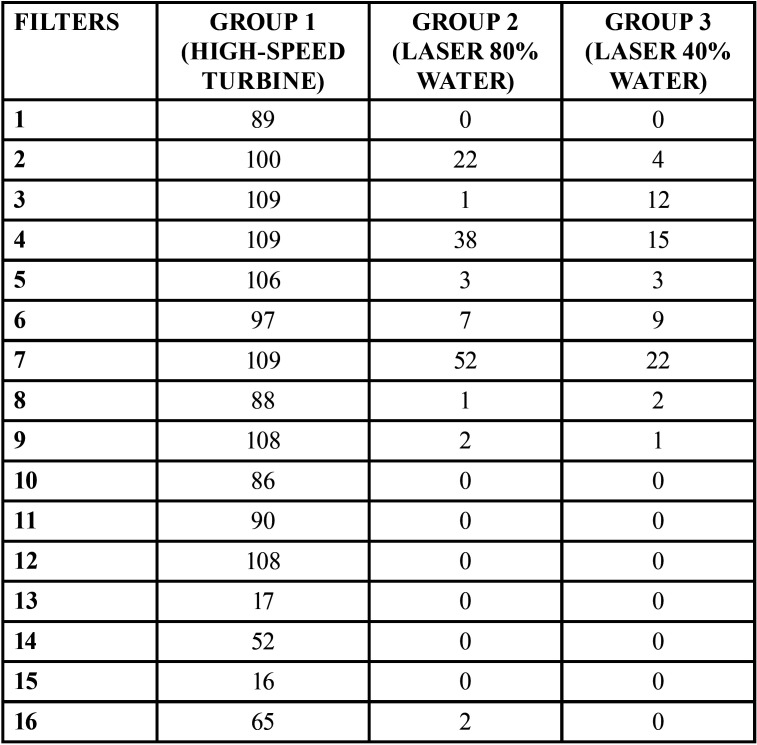
Contaminated area (cm2) in each filter of the 3 groups.

1349 cm2 (77,3%) was detected with fluorescein in group 1, 128 cm2 (7,3%) in group 2 and 68 cm2 (3,8%) in the third group.

The high speed turbine contaminated 70% and 73,5% more surface area of the filters compared to the Laser with 80% and 40% of water respectively.

The difference between the use of different laser parameters resulted in 3.5% less contamination of the total of all filters.

We noted that the mean contamination on the left side of the box was more than on the right side in all groups. In group 1 using air turbine, we measured a mean of 102.6[±7.5 SD] cm2 on the left side compared to 70.6[±32.3 SD] cm2 on the right side. In laser groups 2 and 3, a mean of 12.8[±14.9 SD]cm2 and 6.8 [±5.7SD]cm2 respectively was described on the left side and 0 cm2 of surface contamination on right side.

## Discussion

Splatter and aerosols are differentiated by the particle size, splatter contains fragments larger than 50 μm which rapidly fall down due to the effect of gravity leaving droplet nuclei which can be suspended in air for many hours and can be inhaled into the lungs causing respiratory infection (23). “Bioaerosols” is a more precise term in a clinical setting, as it is always contaminated with blood, tooth and organic particles, bacteria from oral flora or dental plaque and restorative materials (24). Miller S *et al.* (25) in a retrospective analysis for SARS-CoV-2 suggested that airborn transmission is the most likely mechanism rather than only direct or indirect contact with contaminated surfaces. Furthermore, viral RNA in droplets smaller than 5 μm can still be infective (26). Rotary instruments during tooth cutting generate considerable amount of aerosols and the use of abundant water coolant is obligatory (27). Serdar Toroglu *et al.* (28) using the high-speed air-turbine, found a significant increase in enviromental aerosols after five minutes of removing excessive adhesive material after brackets debonding.

Several measures can be taken to reduce the enviromental contamination during these procedures such as pre-procedural mouth wash with 0.2% chlorhexidine which showed significant bacterial reduction in aerosols during ultrasonic scaling (29). The use of rubber dam during tooth preparation and high volume evacuator (HVE) can reduce spread of microorganisms by 90% (12,30). These strategies can be used together or individually.

In recent years, Er,Cr:YSGG laser has become an accepted alternative method for cavity preparation (31), but we didn´t find reports concerning the generation of aerosols using laser cutting compared to conventional high-speed air turbine. Hard tissue laser provides low cutting pressure, less vibration compared to bur cutting and minimal or no need for local anesthesia (32,33). The laser ablation mechanism is achieved through photothermal effect, due to the absorption of laser photons by the water molecule present in the tissue to be cut, producing micro-explosions and surface disruption. Higher water and air ratios reduce the thermal effect (22). In the present study we used two different water percentages to see if it affect the amount of aerosols produced. To avoid an increase in the thermal effect with lower water percentage, we increased the pulse repetition rate and decreased the power, while maintaining air percentage to decrease variables that can affect the dissemination of aerosols (Table 1). However this reduction in power per pulse for group 3 with the double reduction of aerosol may be also due to five times lower energy per pulse, further experimenting in future studies having the same pulse energy but 40% water can be considered, to eliminate possible variables that can alter amount of aerosols.

Conventional air driven handpiece, on the contrary, requires specific air pressure range to function, and air percentage is not a modifiable variable.

Electric handpiece has been introduced to overcome disadvantages of air turbine in terms of maintaining the cutting torque, but there are no studies that describe the aerosols generated. However, it still requires abundant water irrigation due to the heat production (34).

In the present study we observed that higher amount of water can attribute to more splatter and aerosols production. In conventional rotary cavity preparation with air turbine we need more water spray as a coolant than in laser groups to decrease thermal pulp damage. We observed more contamination in the filters located on the left side of the box [1, 2, 3, 4, 5, 6, 7, 16] than those on the right side [8, 9, 10, 11, 12, 13, 14, 15]. This can be attributed to the fact that the operator was right handed orienting the handpiece towards the left side, also the right arm may have blocked the aerosols from reaching the filters located in the right side.

## Conclusions

The contaminated area during the procedure of class I cavity preparation, is reduced by 70% using Er,Cr:YSGG laser compared to high-speed turbine. A slightly higher contamination was observed in 80% versus 40% water laser groups. Further studies are needed with more trials to determine a statistical significant difference between laser and conventional technique, as the use of Er,Cr:YSGG laser could be considered as a safe alternative for aerosols and splatter reduction in daily clinical practice for prevention of airborne diseases transmission in the current pandemic.
